# Improving the use of research evidence in guideline development: 16. Evaluation

**DOI:** 10.1186/1478-4505-4-28

**Published:** 2006-12-08

**Authors:** Andrew D Oxman, Holger J Schünemann, Atle Fretheim

**Affiliations:** 1 Norwegian Knowledge Centre for the Health Services, P.O. Box 7004, St. Olavs plass, N-0130 Oslo, Norway; 2 INFORMA, S.C. Epidemiologia, Istitituto Regina Elena, Via Elio Chianesi 53, 00144 Rome, Italy

## Abstract

**Background:**

The World Health Organization (WHO), like many other organisations around the world, has recognised the need to use more rigorous processes to ensure that health care recommendations are informed by the best available research evidence. This is the last of a series of 16 reviews that have been prepared as background for advice from the WHO Advisory Committee on Health Research to WHO on how to achieve this.

**Objectives:**

We reviewed the literature on evaluating guidelines and recommendations, including their quality, whether they are likely to be up-to-date, and their implementation. We also considered the role of guideline developers in undertaking evaluations that are needed to inform recommendations.

**Methods:**

We searched PubMed and three databases of methodological studies for existing systematic reviews and relevant methodological research. We did not conduct systematic reviews ourselves. Our conclusions are based on the available evidence, consideration of what WHO and other organisations are doing and logical arguments.

**Key questions and answers:**

Our answers to these questions were informed by a review of instruments for evaluating guidelines, several studies of the need for updating guidelines, discussions of the pros and cons of different research designs for evaluating the implementation of guidelines, and consideration of the use of uncertainties identified in systematic reviews to set research priorities.

How should the quality of guidelines or recommendations be appraised?

• WHO should put into place processes to ensure that both internal and external review of guidelines is undertaken routinely.

• A checklist, such as the AGREE instrument, should be used.

• The checklist should be adapted and tested to ensure that it is suitable to the broad range of recommendations that WHO produces, including public health and health policy recommendations, and that it includes questions about equity and other items that are particularly important for WHO guidelines.

When should guidelines or recommendations be updated?

• Processes should be put into place to ensure that guidelines are monitored routinely to determine if they are in need of updating.

• People who are familiar with the topic, such as Cochrane review groups, should do focused, routine searches for new research that would require revision of the guideline.

• Periodic review of guidelines by experts not involved in developing the guidelines should also be considered.

• Consideration should be given to establishing guideline panels that are ongoing, to facilitate routine updating, with members serving fixed periods with a rotating membership.

How should the impact of guidelines or recommendations be evaluated?

• WHO headquarters and regional offices should support member states and those responsible for policy decisions and implementation to evaluate the impact of their decisions and actions by providing advice regarding impact assessment, practical support and coordination of efforts.

• Before-after evaluations should be used cautiously and when there are important uncertainties regarding the effects of a policy or its implementation, randomised evaluations should be used when possible.

What responsibility should WHO take for ensuring that important uncertainties are addressed by future research when the evidence needed to inform recommendations is lacking?

• Guideline panels should routinely identify important uncertainties and research priorities. This source of potential priorities for research should be used systematically to inform priority-setting processes for global research.

## Background

The World Health Organization (WHO), like many other organisations around the world, has recognised the need to use more rigorous processes to ensure that health care recommendations are informed by the best available research evidence. This is the last of a series of 16 reviews that have been prepared as background for advice from the WHO Advisory Committee on Health Research to WHO on how to achieve this.

Providing technical advice to its member states is a core function of the World Health Organization (WHO). Ensuring the quality of the advice that is given is an inherent responsibility of WHO. In this paper we address the following questions related to evaluation of guidelines and their implementation:

• How should the quality of guidelines or recommendations be appraised?

• When should guidelines or recommendations be updated?

• How should the impact of guidelines or recommendations be evaluated?

• What responsibility should WHO take for ensuring that important uncertainties are addressed by future research when the evidence needed to inform recommendations is lacking?

Related questions regarding updating of systematic reviews and implementation are addressed in other articles in this series [1,2].

## What WHO is doing now

The Guidelines for WHO Guidelines suggest that draft guidelines should be subjected to a self-test by the technical development and the steering/liaison groups using a checklist (Table 1) [3].

**Table 1:** Checklist for WHO Treatment Guidelines (from Guidelines for WHO Guidelines [3]).

	Yes	**Questions**	**Reference Points**
**Origin**			
1		Are the Cluster and Department issuing the guidelines clearly identified?	Introduction
**Objective, target audience**			
2		Does the guideline list its objectives, including the patient categories and situation(s) for which the guidelines are intended?	Introduction
3		Does the guideline describe the professional groups to which it is addressed?	Introduction
**Guideline Development Group**			
4		Does the Guideline Development Group include all relevant professional groups, public health experts and end users, including individuals from geographic areas where the guidelines will be applied?	List of members of the guideline development group
5		Does the Group include methodological experts in fields such as search methodology, critical appraisal and cost-effectiveness analysis?	List of members of the guideline development group
**Conflict of interest**			
6		Are all funding sources named, and is there no conflict of interest?	List of funding sources
7		Have all members of the Guideline Development Group and external reviewers declared their interests, and have these interests been recorded in the guideline document?	Annex on documentation of process
8		Does the document describe the method used to minimize any undue influence on the Guideline Development Group and the external reviewers?	Annex on documentation of process
**Evidence**			
9		Was there a systematic comprehensive search for evidence, and has the search strategy been recorded in the guideline?	Annex on documentation of process
10		Has the strength and quality of the evidence on effectiveness been graded?	Annex on documentation of process; evidence table
11a		What percent of recommendations are evidence-based?*	Summary of recommendations
11b		Are the recommendations which are not evidence-based explicitly labeled as "expert opinion" based?	Summary of recommendations
12		Is there explicit consideration of other issues, such as safety and potential misuse in a variety of settings?	Annex on documentation of process; evidence table
13		Is there explicit consideration of issues of cost effectiveness?	Annex on documentation of process; evidence table
14		Is the strength of the recommendation linked to the evidence?	Summary of Recommendations
15		Do the recommendations take into account potential resource constraints?	Implementation issues
**Review**			
16		Were the comments by the external peer review adequately addressed?	Annex on documentation of process
17		Did all members of the Guideline Development Group approve the final document?	Annex on documentation of process
18		Did all members of the Steering Group approve the final document?	Annex on documentation of process
19		Is there a plan for reviewing new evidence and updating the guideline?	Introduction
**Presentation, clarity**			
20		Are the recommendations clearly formulated?	Summary of Recommendations
21		Does the guideline identify and advise on ineffective practices?	Summary of Recommendations
**Implementation plan**			
22		Is there a plan for dissemination and local adaptation of the guideline?	Companion document
23		Are funds available for dissemination and local adaptation for the guideline?	Companion document
24		Are there suggested criteria for monitoring the use in intended settings?	Implementation Issues

This Checklist is intended for the following purposes: (1) As a guide for developing or updating WHO treatment Guidelines. (2) As a check-list for Executive and Regional Directors when giving final approval for publication. To qualify for publication and inclusion in the WHO database of treatment guidelines, a tick mark signifying YES must be placed beside all the 24 criteria, except 11a.

*These are recommendations based on information other than expert opinion.

However, the checklist is not being used and most guidelines appear to be deficient [4]. An unpublished, in house review of WHO guidelines using the AGREE appraisal instrument [5] found that the vast majority of guidelines did not meet most of the AGREE criteria [6]. Although draft guidelines are often sent for peer review, and the Guidelines for WHO Guidelines suggests external review, this is not always done. WHO's Regulations for Expert Committees, in fact, require that "*The expert committee shall draw up and approve its report before the closure of its meeting*." [7]

We are not aware of any assessments of the extent to which WHO guidelines, recommendations or policies are kept up to date or any policies for withdrawing ones that are out of date. The Guidelines for WHO Guidelines offers the following encouragement for undertaking rigorous studies to provide missing evidence, but we are not aware of any other policies linking important uncertainties in guidelines to WHO's priorities for research: *"Sometimes it will be necessary to issue guidelines where no rigorous studies exist, based on the best available evidence. But after issuance of such guidelines, the opportunity could be taken to undertake rigorous studies to provide missing evidence and to evaluate the effectiveness or impact of the guidelines in the actual settings where they are intended to be used. This would allow them to be revised or updated if needed."*

## What other organisations are doing

In a survey of 152 organizations that produce guidelines, technology assessments, or support the use of research in developing health policy a large majority reported using both an internal review process (80%) and external review by experts (82%) [8]. Only 44% reported external review by target users (58% of guideline producers), 43% reported comparing their products with products or input from other groups, and 31% reported using pilot testing. Fifty-two percent of the units that produced guidelines reporting updating them regularly and 45% reported updating irregularly. Thirty-five percent reported collecting data about uptake systematically, and 32% reported systematically evaluating the usefulness or impact of their guidelines in some other way.

In another survey of 18 prominent guidelines development programs, more than half reported monitoring or evaluating the effects of at least some guidelines. All reported using some type of quality system for good guideline development. Eleven used both external and internal review, six used external review only, and one internal review only. In addition seven compared their guidelines with guidelines from other groups and three used pilot testing. All reported updating their guidelines at least occasionally, although only half had formal update procedures.

## Methods

The methods used to prepare this review are described in the introduction to this series [9]. Briefly, the key questions addressed in this paper were vetted amongst the authors and the ACHR Subcommittee on the Use of Research Evidence (SURE). We did not conduct a full systematic review. We searched PubMed and three databases of methodological studies (the Cochrane Methodology Register, the US National Guideline Clearinghouse, and the Guidelines International Network for existing systematic reviews and relevant methodological research that address these questions. The answers to the questions are our conclusions based on the available evidence, consideration of what WHO and other organisations are doing, and logical arguments.

For this review we used articles that we had previously identified, including a review of clinical practice guideline appraisal instruments [10], to locate related articles in PubMed; we searched the National Guidelines Clearinghouse annotated bibliography using the category guideline evaluation with the terms appraisal or impact, and for all categories using updating; and we checked the reference lists of retrieved articles. We also searched for and scanned reviews of methods for setting research priorities that were linked to guidelines development programs by searching PubMed for reviews of research priorities, for articles that addressed both research priorities and practice guidelines, and by searching the Web using Google for sites that addressed methods for setting priorities for research and global research priorities.

## Findings

### How should the quality of guidelines or recommendations be appraised?

Graham and colleagues in a systematic review of instruments for assessing the quality of clinical practice guidelines found 13 instruments published up to 1999 [10]. All instruments were developed after 1992 and contained 8 to 142 questions or statements. Only the Cluzeau instrument, which formed the basis of the AGREE instrument [11,12]' included at least one item for each of the 10 attributes that the authors identified across instruments. This instrument and that of Shaneyfelt and colleagues [13]. were the only instruments that had been validated. They concluded that there was insufficient evidence to support the exclusive use of any one instrument, although the AGREE instrument has received the most evaluation. Vlayen and colleagues updated the review by Graham and colleagues up to 2003 [14]. They found 24 different appraisal tools. The 24 instruments included a total of 469 questions that they also grouped into 10 dimensions: validity, reliability/reproducibility, clinical applicability, clinical flexibility, multidisciplinary process, clarity, scheduled review, dissemination, implementation, and evaluation. They found three instruments that addressed all 10 dimensions and three additional instruments based on the Cluzeau instrument, one of which, the AGREE instrument, was the only one to have been validated. They found that the AGREE instrument was a validated, easy-to-use, and transparent instrument, which was internationally developed and widely accepted, but noted two limitations that they considered important: although it can be used to compare clinical practice guidelines, it does not set a threshold to classify them as good or bad, and it does not assess the quality of the evidence supporting the recommendations.

The AGREE instrument was developed through a process of item generation, selection and scaling, field-testing and refinement [5]. The final version of the instrument contained 23 items grouped into six domains: scope and purpose, stakeholder involvement, rigour of development, clarity and presentation, applicability, and editorial independence.

### When should guidelines or recommendations be updated?

Shekelle and colleagues, based on a review of 17 guidelines published by AHRQ, estimated that no more than 90% were still valid after 3.6 years and that about half the guidelines were outdated in 5.8 years. They recommend that guidelines should be reassessed every three years, based on the lower 95% confidence interval for their estimate of when one of ten guidelines would no longer be up-to-date. They suggest several ways of expeditiously assessing the need for updating guidelines including conducting limited searches by groups that are familiar with the topic, such as Cochrane review groups, focusing searches on research that the guidelines panel considered would play a pivotal role in requiring revision of the guideline, periodic review of the guidelines by experts not involved in developing the guidelines, and considering guidelines development as an ongoing process, rather than a discreet event, with members of guideline panels serving fixed periods with a rotating membership.

Gartlehner and colleagues compared the approach suggest by Shekelle and colleagues of a limited search using review articles, commentaries and editorials, to a conventional process using typical systematic review methods in terms of comprehensiveness and effort [15]. They applied both approaches independently to assess the need to update six topics from the 1996 Guide to Clinical Preventive Services from the US Preventive Services Task Force [16]. They found that although the limited search approach identified fewer eligible studies than the traditional approach, none of the studies missed was rated as important by task force members acting as liaisons to the project with respect to whether the topic required an update. On average, this approach produced substantially fewer citations to review than the traditional approach. The effort involved and potential time saving depended largely on the scope of the topic. They found that involving experts in assessing how current the guidelines were was not helpful, in contrast to Shekelle and colleagues.

Johnston and colleagues found that an updating strategy for cancer practice guidelines identified 80 pieces of new evidence over a one-year period relating to 17 of 20 guidelines [17]. They found on average four pieces of new evidence per guideline, but there was considerable variation across the guidelines. Of the 80 pieces, 19 contributed to modifications of clinical recommendations in six practice guidelines, whereas the remaining evidence supported the original recommendations. Their updating process yielded important findings, but was resource intensive. They found that it would be possible to reduce the scope of the sources searched routinely to MEDLINE, the Cochrane Library and meeting proceedings.

The findings of these three studies of the need to update guidelines is consistent with findings from studies of the need to update systematic reviews, which generally support the conclusion that in situations where time or resources are limited, thorough quality assessments should likely take precedence over extensive literature searches [1].

### How should the impact of guidelines or recommendations be evaluated?

Strategies ranging from passive dissemination to intensive, complex interventions have been used to implement guidelines and a range of study designs has been used to evaluate the impact of these strategies using a range of outcome measures [18,19]. Passive strategies have often not been effective, however there is limited evidence to support decisions about which guideline dissemination and implementation strategies are likely to be efficient under different circumstances [2,18].

Study designs that can be used to evaluate the impact of guidelines include randomised designs, particularly cluster randomised trials, a range of observational study designs, including interrupted time series analyses, controlled before-after studies and uncontrolled before-after studies [20]. The advantage of using randomised designs for impact assessments is that they give greater confidence that the measured impact of a program is attributable to whatever implementation strategy was used and not to some other factor [21-23]. It is generally not possible to predict differences in the size, or even the direction, of estimates of treatment effects for the same intervention when it is generated in randomized and non-randomized studies [22]. There have been similar findings for impact evaluations of development programs [21] and implementation strategies. For example, a systematic review of continuous quality improvement found improvements in 41 of 43 single site before-after studies and most of 13 multi site before-after studies, but no improvements in three randomised trials [24].

A wide variety of techniques to gather data have been used singly or in combinations, including questionnaires, interviews, observation, audit and using routinely collected data. Self-report may not be consistent with more objective measures of practice. Collecting reliable data in low and middle-income countries (LMIC) can be a major challenge, where available records and routinely collected data may be lacking. We did not find any systematic reviews of strategies for collecting data for impact evaluations in LMIC.

### What responsibility should WHO take for ensuring that important uncertainties are addressed by future research when the evidence needed to inform recommendations is lacking?

Priority-setting exercises for global health research have used various methods and processes [25]. We have not found examples of priority setting programs based on important uncertainties identified in guidelines. A number of exercises have, however, used systematic reviews to inform priority-setting processes [26,27]. A comparison of four sources of potential priorities for the NHS Health Technology Assessment Programme found that a widespread consultation of healthcare commissioners, providers and consumers was the largest source of suggestions, but the success rate of this source, in terms of being commissioned, was low. Research recommendations from systematic reviews provided the second largest source of priorities and the best success rate of all sources.

## Discussion

There are at least 24 different instruments available for assessing the quality of clinical practice guidelines. We did not find similar tools developed for assessing the quality of public health or health policy recommendations, although the domains that are addressed by clinical practice guidelines appraisal instruments are applicable to public health and health policy recommendations.

Up to now, self-assessment of guidelines using the Guidelines for WHO Guidelines checklist has not been successful. Moreover, most guidelines programmes rely on external review, as well as internal review. WHO should put into place processes to ensure that both internal and external review of guidelines is undertaken routinely using appropriate criteria.

Processes should also be put into place to ensure that guidelines are monitored routinely to determine if they are in need of updating. To ensure that this is done as expeditiously as possible, people who are familiar with the topic, such as Cochrane review groups, should conduct limited searches routinely. Guideline panels should identify research that would require revision of the guideline and searches should focus particularly on this research. Periodic review of guidelines by experts not involved in developing the guidelines should also be considered, and consideration should be given to establishing guideline panels that are ongoing with members serving fixed periods with a rotating membership.

Recommendations may need to be adapted to specific settings, can only be implemented in specific settings, and their impact can only be assessed in specific settings. WHO headquarters and regional offices, however, should support member states and those responsible for deciding and implementing policies to evaluate the impact of their policies by providing advice regarding impact assessment, practical support and coordination of efforts. Before-after evaluations should be used cautiously, if at all, and when there are important uncertainties regarding the effects of a policy or its implementation, randomised evaluations should be used when possible.

Guideline panels should routinely identify important uncertainties and research priorities. This source of potential priorities for research should be used systematically to inform priority-setting processes for global research.

## Further work

Work is needed to ensure that the AGREE instrument or a similar instrument is suitable for assessing the broad range of guidelines, recommendations and policies that WHO produces. In particular, its suitability for assessing public health and health policy recommendations should be assessed. Additional items should also be added to address concerns about equity, which are not currently addressed in the AGREE instrument, and considerations that are specific to guidelines that are developed internationally rather than in a specific country or setting. Work is also needed on developing practical methods to collect reliable data that can be used in impact evaluations in LMIC.

## Competing interests

ADO and AF work for the Norwegian Knowledge Centre forthe Health Services, an agency funded by the Norwegian government that produces systematic reviews and health technology assessments. All three authors are contributors to the Cochrane Collaboration. ADO and HJS are members of the GRADE Working Group. HJS is documents editor and chair of the documents development and implementation committee for the American Thoracic Society and senior editor of the American College of Chest Physicians' Antithrombotic and Thrombolytic Therapy Guidelines.

## Authors' contributions

ADO prepared the first draft of this review. HJS and AF contributed to drafting and revising it.
